# Exploring the Immunoproteome for Ovarian Cancer Biomarker Discovery

**DOI:** 10.3390/ijms12010410

**Published:** 2011-01-14

**Authors:** Karina Martin, Carmela Ricciardelli, Peter Hoffmann, Martin K. Oehler

**Affiliations:** 1 Adelaide Proteomics Centre, School of Molecular and Biomedical Science, University of Adelaide, Adelaide, Australia; E-Mails: karina.martin@adelaide.edu.au (K.M.); peter.hoffmann@adelaide.edu.au (P.H.); 2 Research Centre for Reproductive Health, School of Paediatrics and Reproductive Health, Robinson Institute, University of Adelaide, Adelaide, Australia; E-Mail: carmela.ricciardelli@adelaide.edu.au; 3 Department of Gynaecological Oncology, Royal Adelaide Hospital, Adelaide, Australia

**Keywords:** ovarian cancer, autoantibodies, immunoproteomics

## Abstract

Most scientific efforts towards early detection of ovarian cancer are commonly focused on the discovery of tumour-associated antigens (TAA). Autologous antibodies against TAA, however, may serve as more sensitive diagnostic markers. They circulate in the blood before TAA and are usually more abundant than the TAAs themselves as a result of amplification through the humoral immune response. Accumulating evidence also suggests that a humoral response already exists during malignant transformation when aberrant gene expression is translated into premalignant cellular changes. This article reviews the current knowledge about autoantibodies against TAA in ovarian cancer and presents current immunoproteomic approaches for their detection.

## 1. Introduction

Ovarian cancer is the leading cause of death from gynaecological malignancies [1,2]. It accounts for 5% of all cancer deaths among women with an estimated 21,880 new cases and 13,850 deaths from ovarian cancer in the United States in 2010 [2]. The poor prognosis and high mortality rate associated with the disease have not significantly improved over the last 30 years despite advances in treatment [3]. This arises as ovarian cancer development is largely asymptomatic resulting in the majority of patients (62%) presenting with advanced disease (FIGO stage III and IV) [2]. Current therapies prove effective for patients with early stage disease (FIGO stage I/II) where 5-year survival rates range from 73% to 93%. Their usefulness, however, is limited for patients with advanced stage disease where the 5-year survival is only about 30% [2,4]. To date transvaginal ultrasonography (TVU) and serum levels of the cancer antigen 125 (CA125) are used alone or in combination to diagnose ovarian cancer. However, both approaches have limitations that render them inappropriate for screening the general population.

TVU has poor diagnostic power where a sensitivity of 84.9%, specificity of 98.2% as well as positive predictive value (PPV) of 5.3% for primary ovarian and tubal cancer have been reported in an ongoing ovarian cancer screening study [5]. Due to the low lifetime risk of an individual woman developing ovarian cancer in the population (1.4%) the minimum requirements for a global screening strategy to detect early ovarian cancer are benchmarked at >75% sensitivity and 99.6% specificity [6,7]. These values for sensitivity and specificity aim to generate a PPV of 10% where no more than 10 suspected individuals would need to undergo surgery to confirm one ovarian cancer case. Due to potential complications associated with this surgery a PPV less than 10% has been deemed unacceptable.

CA125, a large glycoprotein from the mucin family, was identified by Bast and colleagues in 1981 [8]. They subsequently demonstrated that serum CA125 levels over 35 U/mL discriminate between healthy and disease cases [9]. Although effective at identifying 80% of patients with late stage disease, CA125 is only elevated in less than 50% of early stage I/II ovarian cancer [10,11]. Another caveat to the use of CA125 is its elevation in benign conditions including endometriosis, fibroids, pelvic inflammatory disease as well as various other malignancies. Consequently the usefulness of CA125 as an effective screening marker for identifying early stage disease is limited. Therefore novel ovarian cancer biomarkers with high specificity and sensitivity are warranted.

## 2. Biomarkers

To date over thirty biomarkers for ovarian cancer have been reported in the literature (reviewed [6,12]). The vast majority are proteins produced by the cancer which can be identified in the sera of patients. A major limitation of these proteins as biomarkers is the inability to detect them at early stages of cancer development. This arises due to low amounts of the protein being secreted by small early stage cancerous lesions and the high dynamic range of proteins in serum (12 orders of magnitude) [13].

Autologous antibodies against tumour associated antigens (TAAs) may serve as more sensitive diagnostic markers. TAAs are tumour specific proteins and peptides that are subject to dysregulation, mutation or post translational modification (PTM) during cancer development and have been reported as potential causes of an (auto-)antibody response [14]. Antibodies to TAA have three qualities which make them ideal candidates for biomarker validation and screening. Firstly, they are detectable at early stages of disease. Their production by B-lymphocytes can be activated by a single antigen resulting in signal amplification through the humoral immune response. Secondly, autoantibodies are naturally resistant to proteolysis and metabolism experienced by other molecules, attributing to their long half-life of approximately 21 days [15–17]. This stability allows their reliable detection and facilitates their use in the development of diagnostics. Finally, autoantibodies are present in the sera of patients, an accessible biological material, and can therefore be analysed through well established techniques [13].

Various methods like serological analysis of recombinant cDNA expression libraries (SEREX), phage display, protein microarray, serological proteome analysis (SEPRA) and immunoaffinity chromatography have been used to identify autoantibody biomarkers in various malignancies. However, reports on the use of these technologies in ovarian cancer are limited.

### 2.1. Serological Analysis of Recombinant cDNA Expression Libraries (SEREX)

**Figure 1:**
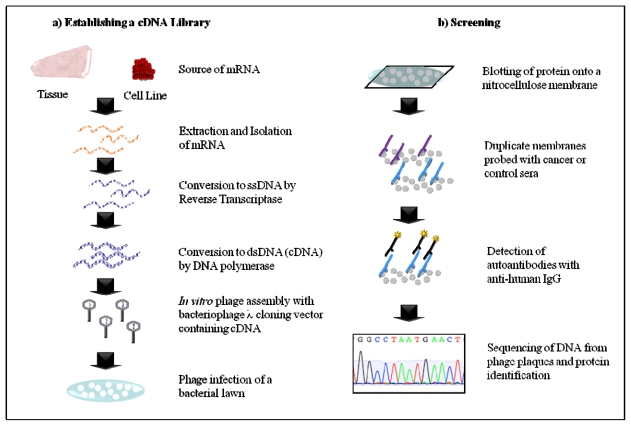
Schematic outlining autoantibody identification by SEREX. (**a**) mRNA extracted from cancer tissue or cell line is converted to cDNA prior to cloning into a phage vector, which is packaged into phage virions and expressed during bacterial infection; (**b**) Proteins generated during lytic infection are blotted onto a membrane and probed with sera. Upon detection of cancer specific autoantibody signals phage DNA is sequenced and TAA identified through a database search.

SEREX utilises cDNA libraries for the expression and detection of antigens that elicit a humoral immune response in patients. To this end, mRNA extracted from cancer tissue or a tumour cell line is converted to cDNA by *in vitro* methods and subsequently cloned into a bacteriophage for infection of *Escherichia coli (E. coli)* (Figure 1a). During lytic infection the recombinant proteins are expressed and can be blotted onto a nitrocellulose membrane for antibody screening with sera (Figure 1b). Seroreactive proteins can then be identified by sequencing the phage cDNA from positive plaques. SEREX has been useful for identifying several tumour specific antigens that generate a humoral immune response in cancers such as those from the kidney, lung, breast and colon [18]. However, this approach has inherent limitations that restrict the types of TAAs that may be identified to those that can be expressed in a prokaryotic system. This precludes TAAs that require folding mechanisms unique to eukaryotes to achieve the correct conformational epitope for recognition and those that are subject to PTM, which is a common property of cancer antigens. Furthermore, the TAA encoded in the cDNA library may not have the full length protein sequence. Thus those patients that elicit a humoral immune response to different antigenic determinants of the same TAA may be missed using this system. Furthermore, identification of TAAs is limited to those that are expressed by the patient tumour or cell line in which the cDNA library was derived. As the majority of cancers are very heterogeneous more than one cDNA library may be required to identify a comprehensive set of seroreactive TAAs [19]. Finally, the generation and screening of a cDNA library is labour-intensive, not amenable to automation and therefore presents as challenge for high-throughput analysis.

A study by Stone and colleagues applying SEREX screening to advanced stage ovarian cancer patients identified 25 antigens inducing a humoral immune response [19]. The majority of TAAs were recognised only by autologous serum, however 6 antigens were found to be immunogenic in at least 2 of the 25 patient sera screened. A secondary screening using 25 allogenetic sera showed that only 36% (9/25) of patients demonstrated immunity against at least one of the 25 TAAs in the panel. Here, only 7 TAAs (Table 1) were found to generate an autoantibody response in at least 1 of those 9 patients. As these autoantibodies were not present in the 45 healthy controls they were thought to have potential as diagnostic indicators of ovarian cancer. However, further analyses in a larger cohort of ovarian cancer patients are required.

Lou and co-workers screened a commercially available ovarian carcinoma cDNA library with ascites pooled from 5 advanced stage ovarian cancer patients [20]. Twelve novel immunoreactive tumour antigens were identified (Table 1). Autoantibodies against one antigen—HSP90—were further assessed by ELISA. At a fluorescence ratio cut-off of 2.0 no healthy individuals and only 5% of individuals with benign gynaecologic disease demonstrated immunity against HSP90, suggesting that HSP90 autoantibodies may reflect a specific response to the cancer. Prevalence of HSP90 autoantibodies was higher in advanced stage (32%) than early stage ovarian disease (10%). It is unclear from this small patient cohort if HSP90 autoantibodies would be suitable for early detection. Results from a larger cohort are required.

Serological screening of a commercially available cDNA library by Lokshin and colleagues identified 20 TAAs of which 14 were previously unreported [21]. Amongst these interleukin-8 (IL-8) and the corresponding autoantibody were subsequently examined as potential biomarkers. As the average serum levels for IL-8 autoantibodies were significantly lower in healthy individuals compared to both early stage and late stage ovarian cancer patients it was concluded that they might have potential diagnostic value. Receiver operating characteristic (ROC) curves generated from the early stage ovarian cancer patient cohort demonstrated IL-8 autoantibodies to have a similar sensitivity (65.5%) to that of IL-8 (62.6%) at 98% specificity. Consequently, 79–80% of patients were correctly identified by IL-8 or IL-8 autoantibodies. Unfortunately, the mean serum levels for IL-8 autoantibodies in patients with benign gynaecological disease were not significantly different from those with ovarian cancer. As a result IL-8 autoantibodies alone were not specific enough for screening of early disease. However, the utility of IL-8 autoantibodies as a complementary marker to CA125 was promising. A combination of the three biomarkers, IL-8, IL-8 autoantibodies and CA125 resulted in an increase in the sensitivity to 87.5% compared to CA125 alone (76.8%) without compromising specificity (98%). Thus, IL-8 autoantibodies had diagnostic potential when incorporated into a panel of ovarian cancer biomarkers, however these need to be investigated in larger cohorts.

With the aim to identify new therapeutic targets Jin and colleagues performed autologous screening of a cDNA library created from a single ovarian cancer patient [22]. Of the 27 seroreactive peptides identified 7 were classified as proteins transcribed from expressed sequence tags (EST). EST 1753 generated protein, referred to as OVA66, was assessed for immunogenicity by ELISA using 48 control sera and 113 cancer sera from patients with various malignancies including ovarian cancer (24%). Although the difference in OV66 levels were significantly different between cancers and controls, OVA66 autoantibodies expression was not restricted to ovarian cancer patients. Autoantibodies were detected in 52.6% hepatocellular carcinoma patients, 27.3% colon cancer patients, 23.8% gastric cancer patients compared to 22.2% of ovarian cancer patients. Therefore, the TAA OVA66 would not be useful as specific ovarian cancer biomarker.

In conclusion, to date SEREX has enabled the identification of several TAAs that elicit a humoral immune response in patients with ovarian cancer [23]. However, further studies are required to determine their diagnostic value.

### 2.2. Phage Display

**Figure 2:**
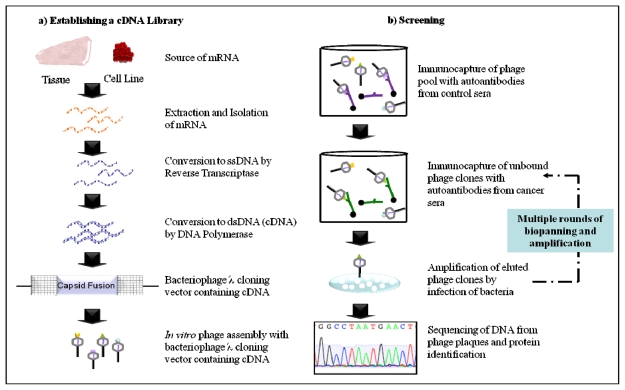
Schematic outlining autoantibody identification by phage display. (**a**) mRNA extracted from cancer tissue or cell line is converted to cDNA. Phage vectors encoding human cDNA sequences are assembled into virions for expression as recombinant capsid fusion proteins; (**b**) Phage clones presenting immunoreactive peptides are immunoprecipitated from the pool with antibodies from the sera of healthy individuals. Unbound clones are immunoprecipitated with antibodies from the sera of cancer patients, eluted and amplified through infection of *E. coli*. Phage clones isolated by multiple rounds of immunoprecipitation with antibodies from cancerous sera are then sequenced and antigenic proteins identified.

In a phage display proteins are expressed as fusions of the viron capsid proteins, thus eliminating the need for infection of bacteria for protein production like in SEREX (Figure 2a). Phage clones with seroreactive surface proteins are selected by incubating the pool with autoantibodies bound to protein G-sepharose. Routinely, phage clones that are recognised by autoantibodies in the sera of healthy individuals are depleted from the pool before repetitive biopanning with autoantibodies in the sera of cancer patients (Figure 2b). This ensures that phage clones identified have a cancer specific immune response. Although this approach enables extensive identification of cancer autoantibodies it has the same limitations encountered by SEREX: Proteins expressed are restricted to those that are found in the source of the cDNA library, can be expressed by phage and lack PTMs or conformational epitopes.

Vidal and co-workers utilised a phage display library containing 10^8^–10^9^ peptide sequences to screen for autoantibodies in the ascites of a patient with stage IV papillary serous ovarian carcinoma [24]. Peptide sequence CVPELGHEC was found to be displayed by 86% of the phage clones isolated in the screening and was subsequently assessed for immunogenicity by ELISA. Patients with metastatic gastrointestinal cancer, non-malignant liver cirrhosis, benign gynaecological disease and healthy individuals all demonstrated low autoantibody reactivity of less than 14%. Although 58.8% of stage IV ovarian cancer patients analysed had anti-CVPELGHEC autoantibodies, only 7.1% of patients with stage III ovarian cancer demonstrated reactivity. These findings suggest that autoantibodies to CVPELGHEC would not be a useful biomarker due to a high false negative rate.

Chatterjee and colleagues utilised phage display in conjunction with protein microarray to identify novel antigens and validate autoantibodies present in the sera of patients as biomarkers for ovarian cancer [25]. Upon biopanning of a phage display cDNA library 480 positive plaques were identified. These plaques were subsequently screened with sera from patients with ovarian cancer, borderline tumours, benign gynaecological disease, endometrial cancer and healthy controls. Only 45 of the antigens were identified as generating a specific immune response in ovarian cancer patients. A panel of the 6 best clones, as determine by ROC curve analysis, demonstrated an average sensitivity and specificity of 32% and 94%, respectively. Upon sequencing of the phage cDNA clones these 6 antigens were not natural gene products but formed a novel sequence when recombinantly expressed as a phage fusion protein. Termed mimotopes, it was proposed that these fusion proteins demonstrate seroreactivity in patients due to molecular mimicry of the epitopes of native proteins that have elicited the immune response. These mimotopes account for approximately 80% of the antigens identified by this approach and may have clinical utility as antigens for autoantibody screening for ovarian cancer. The remaining 10 antigens (Table 1) that represent native gene products have been reported in other malignancies and therefore an autoantibody response to these proteins as ovarian cancer specific biomarkers requires further validation.

Phage display has enabled the identification of various antigens that elicit autoantibody responses in ovarian cancer. However the majority of those are mimotopes of native proteins. Thus the identity of those antigens may need to be determined before the corresponding autoantibodies are investigated as biomarkers.

### 2.3. Protein Microarray

**Figure 3:**
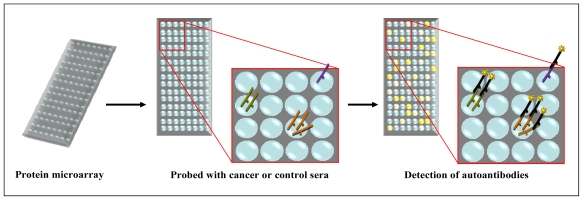
Schematic outlining autoantibody identification by reverse-phase protein microarray. Microarray slides spotted with recombinant proteins are incubated with sera from cancer patients or healthy controls. Autoantibodies captured by proteins present in the array are visualised through incubation with fluorophore conjugated anti-human IgG secondary antibody. Differential signals between cancer and control protein microarrays are subjected to statistical analysis to identify autoantibodies specific for cancer.

Protein microarrays enable the identification of protein-protein interactions, such as antibody-antigen binding, in a high-throughput and automated setting (Reviewed [32,33]). The two types used to explore the immunoproteome are termed forward-phase and reverse-phase microarray depending on the nature of the capture/bait molecule. Reverse-phase microarrays employ the antigenic nature of proteins (the bait) to capture antibodies (the prey). The source of the bait may be from a commercial recombinant protein library arrayed onto a slide (Human ProtoArray, Invitrogen, Carlsbad, CA) [34], cell free cDNA expression and protein immobilisation onto a slide [35] or lysates from cancer tissue or cell lines [36–38]. In the case of cell lysates, proteins require liquid phase fractionation, incorporating isoelectric focusing and reverse-phase liquid chromatography (LC), prior to printing onto an array support. The array can then be probed with patient or control sera in a multiplexed approach followed by incubation with fluorophore conjugated anti-human IgG secondary antibody (Figure 3). Immunoreactive fractions can be subsequently detected and data analysed. This technology can be used to identify novel TAAs or validate known TAAs by screening the sera from several patients.

Forward-phase microarrays utilise immobilised antibodies (bait) to capture TAAs (prey). Immobilisation of antibodies to a range of support medias can involve covalent linking (aldehyde), adsorption (poly-L-lysine), affinity interaction (biotin-streptavidin, Protein G) and capture (agarose, polyacrylamide) that vary in binding capacity and effect on antibody conformation/activity [39]. Using in house or commercially available monoclonal antibody microarrays (BD Clontech AB Microarray 500, Mountain View, CA) TAAs from tumour cell lysates are captured and detected by sandwich ELISA. Using this approach Qin and coworkers demonstrated that novel TAAs specific for prostate cancer could be identified using purified and labelled autoantibodies from serum [40]. However, the use of two antibodies that recognise different epitopes of a single antigen is required. Alternatively, cancer and control tissue lysates can be fluoresently labelled for direct detection of an interaction. In both protein microarray approaches immunoreactive antigens can be subsequently identified by MS. To date only reverse-phase protein microarrays have been employed to identify novel TAAs or characterise known TAAs and their respective autoantibodies in ovarian cancer.

Gnjatic and colleagues used a commercially available protein microarray (V4.0, Invitrogen, Carlsbad, CA) with 8277 human proteins to identify autoantibodies in ovarian cancer sera [34]. Of the arrayed proteins, 197 demonstrated a greater seroreactivity and stronger fluorescent signal in the patient cohort compared to the healthy controls. The authors note that further studies are ongoing to elucidate the diagnostic potential of these autoantibodies.

An earlier study by Hudson *et al.* similarly used the ProtoAray human protein microarray (V3.0, Invitrogen) to identify seroreactive antigens in ovarian cancer [26]. At that time only 60% of the recombinant proteins of the currently available array were present. Ninetyfour TAAs were identified as having immunogenicity specific to ovarian cancer patients when compared to healthy individuals, some of which were not identified in the study by Gnjatic and others [34]. However, no further exploration of the autoantibodies to these TAAs has been carried out to date.

The same commercial protein microarray (V4.0, Invitrogen, Carlsbad, CA) was used to screen a pool of ascites samples from 30 patients with serous ovarian carcinoma by another group [27]. Here, only 10 proteins were identified as having seroreactivity specific for ovarian cancer. Of the identified proteins L-aminoadipate-semialdehyde dehydrogenase-phosphopantetheinyl transferase (AASDHPPT) was found to have the greatest signal intensity compared to controls. However, upon further analysis of ascites from 100 patients by ELISA, anti-AASDHPPT autoantibodies at a very high titre were only present in a single patient that was used in the preliminary screening. For this reason pooling of samples is controversial [41,42].

Conversely, Taylor and co-workers used a dot-blot protein array to define the immunogenicity of 12 previously identified autoantigens in ovarian cancer patients (Table 1) [28]. Of the 12 TAAs analysed the mean autoantibody level against nucleophosmin, cathepsin D and SSX common antigen was significantly greater in patients at each stage (I–IV) compared to healthy or benign controls. All other autoantibodies assayed were able to discriminate between cancer patients and healthy controls to some extent. Further analysis was therefore performed to determine the specificity of the immune response against these antigens in ovarian cancer compared to other malignancies. Of the 6 autoantigens analysed placental-type alkaline phosphatase (PLAP) autoantibodies demonstrated the greatest specificity for ovarian cancer, however immunoreactivity in stage I patients was not significantly different to healthy or benign controls. It was therefore concluded from this study that a two-tiered approach should be taken. Firstly a panel of autoantigens such as nucleophosmin, cathepsin D and SSX common antigen, characterised in this study, would be employed to discriminate between cancer patients and those with benign gynaecological disease. Secondly, subsequent testing for ovarian cancer by means of specific marker(s) such as PLAP would follow.

Overall protein microarray is a promising technique that enables the identification and characterisation of various autoantibodies specific to ovarian cancer. However, most of the autoantibodies identified have not been validated regarding their diagnostic value.

### 2.4. Serological Proteome Analysis (SEPRA)

**Figure 4:**
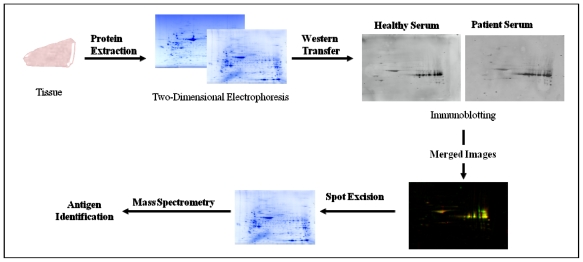
Schematic outlining autoantibody identification by SEPRA. Cancer tissue lysates are subjected to two-dimensional electrophoresis prior to electrophoretic transfer onto a low fluorescent PVDF membrane. Serum from cancer patients or healthy controls is used to probe the membrane and captured autoantibodies detected by incubation with fluorophore conjugated anti-human IgG secondary antibody. Blots are imported into computational software where they can be viewed in multichannel mode and merged to enable identification of immunoreactive spots specific to cancer. Protein spots of interest are excised from a replicate gel and analysed by mass spectrometry for identification.

Serological proteome analysis utilises the separation of proteins by two-dimensional gel electrophoresis (2-DE) combined with Western blotting to screen patient sera for autoantibodies against cancer specific antigens (Figure 4). 2-DE is a classical proteomic technique that enables the separation of proteins in a complex mixture based on charge and molecular weight (Mr). Separation in the first dimension exploits the acidic or basic properties of proteins, which is based on the content of positively or negatively charged amino acid side chains. Under an applied electric field proteins will migrate in a pH gradient until they reach their isoelectric point (p*I*) where the sum charge equals zero [43]. In the second dimension, proteins become separated based upon their molecular weight using conventional SDS-polyacrylamide gel electrophoresis (SDS-PAGE), where low molecular weight proteins migrate to the anode more rapidly than heavier proteins.

Proteins and peptides isolated from cancer cell lines or tumour tissue are separated by 2-DE, electrophoretically transferred onto a membrane and subsequently probed with patient or control sera for biomarker discovery and validation. Autoantibodies bound to antigens can then be detected by probing the membrane with a secondary antibody that is raised against the fragment crystallisable (Fc) region of human IgG. The secondary antibody is typically linked to an enzyme, such as horse radish peroxidase (HRP), which catalyses a chemoluminescent reaction in the presence of substrate that can be visualised on X-ray film. Alternatively, the secondary antibody may be conjugated to a detectable fluorescent tag (Cy5, Cy3, *etc.*) that can be visualised by scanning of the membrane with a fluorescence scanner. Seroreactive proteins specific to cancer can then be identified by mass spectrometry (MS).

SEPRA, in contrast to SEREX and phage display, enables the detection of proteins that have undergone PTMs and allows the assessment of several 1000 proteins simultaneously on a single format under defined conditions [44]. Limitations of detecting autoantibodies by SEPRA stem from the inherent limitations of 2-DE. Those are the potential loss of small (<15 kDa), very large (>200 kDa), very acidic (p*I* < 3), very basic (p*I* > 10) and very hydrophobic proteins as well as the inability to detect TAA with conformational epitopes due to the denaturing conditions.

Several reports have been published using SEPRA for successful identification of autoantibodies against TAAs in cancers of the kidney [45], lung [46], stomach [47], breast [48,49], pancreas [50] and other organs [51]. The first study using SEPRA to investigate the presence of ovarian cancer specific autoantibodies and their potential as ovarian cancer biomarkers was published by Barua *et al* [52]. Overall, the level of autoantibodies detected in sera was significantly higher for ovarian cancer patients compared to controls. While a similar proportion of sera samples from cancer patients reacted against healthy tissue protein lysates (81%) as well as tumour lysates (69%), spot differences in two-dimensional Western blots demonstrated the presence of unique cancer antigens. Although this principle had been previously established for several other cancers [53], this study demonstrated that there are ovarian cancer specific autoantibodies that may have biomarker potential. However, the antigens were not identified in this study and to date there are no further reports on the identification of biomarkers for ovarian cancer by SEPRA.

### 2.5. Immunoaffinity Purification Methods

**Figure 5:**
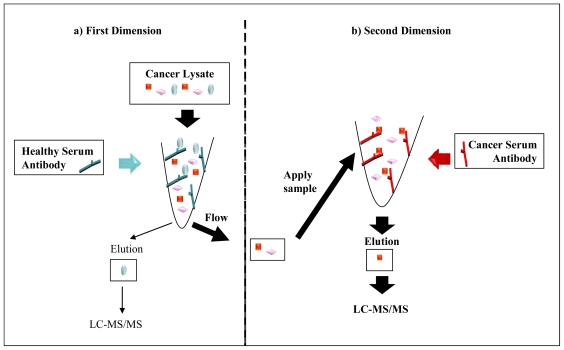
Purification of TAAs from cancer tissue lysates using two dimensional affinity chromatography. (**a**) Autoantigens that are not specific to cancer (blue circles) are captured in the first dimension by autoantibodies from healthy subjects; (**b**) Proteins that are not captured in the first dimension (flow through; square and diamond) are subjected to the second dimension where cancer specific TAAs will be captured (square) by autoantibodies from cancer patients. The subsequent elution fractions from both columns are assessed by LC-MS/MS. LC: liquid chromatography; MS: mass spectrometry.

Affinity purification has been widely used to enrich targeted proteins of interest from a complex sample. Recently, purified autoantibodies from the sera of cancer patients or healthy controls have been used to generate immunoaffinity columns that capture autoantigens from cancer tissue lysates [54–56]. Termed multiple affinity protein profiling (MAPPing), this approach first utilises autoantibodies from healthy individuals to capture autoantigens that are present in cancer tissue lysates (Figure 5a). This first dimensional separation effectively removes autoantigens that do not elicit a cancer specific immune response but are present in the healthy population. Unbound proteins are then applied to an immunaffinity column created with autoantibodies from cancer patients (second dimension) (Figure 5b). Captured TAAs are subsequently eluted and identified by online tandem MS. This two-dimensional chromatography based separation ensures that the identified antigens reflect a cancer specific immune response and identify autoantibodies present in patient sera which are potential biomarkers. Caron and colleagues [54] developed this technique to identify autoantibodies as biomarkers for breast and colon cancer. However, since published in 2007, no other group has described the use of this approach for cancer biomarker discovery.

**Figure 6:**
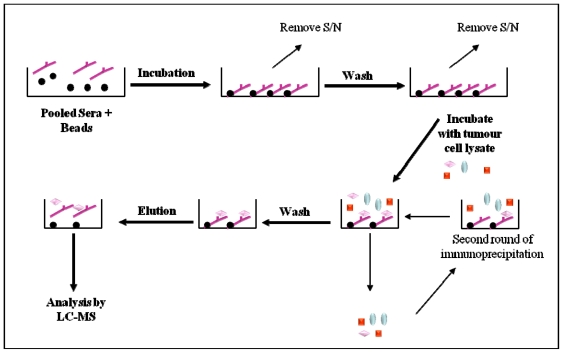
Immunoprecipitation method employed by Philip *et al.* [29] to identify ovarian cancer specific TAAs. Pooled healthy or cancer sera from patients were incubated with protein A/G sepharose beads before incubation with lysates from an ovarian cancer cell line. Co-eluted autoantibodies were separated from the autoantigens by liquid chromatography (LC) and were subsequently identified using mass spectrometry (MS). S/N: supernatant.

Immunoprecipitation of cancer lysates using autoantibodies acquired from serum of cancer patients and healthy individuals has also been explored. Philip [29] incubated lysates from two ovarian adenocarcinoma cell lines with pooled antibodies from cancer or control sera that are coupled to protein A/G beads (Figure 6). Following immunocapture and elution of antigens, samples were fractionated and analysed by LC-MS/MS. Several autoantigens and their respective autoantibodies, specific to ovarian cancer, were identified. Of those, eight autoantigens were found to be precipitated from all 5 different pools of cancer sera (Table 1). Although promising, no further analysis was performed to determine the prevalence or diagnostic value of these autoantibodies.

**Figure 7:**
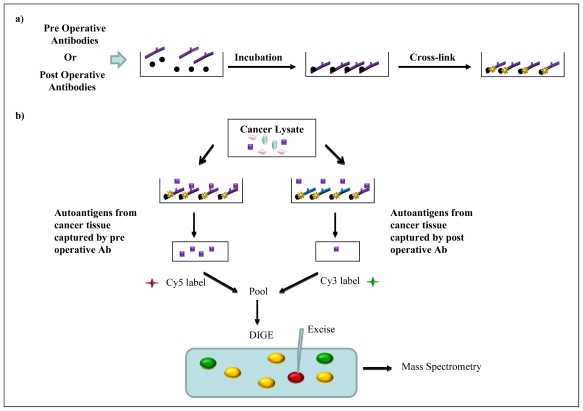
Immunoprecipitation method employed by Gagnon *et al* [30] to identify autoantigens and their respective autoantibodies in ovarian cancer patients. (**a**) Pre or post operative serum antibodies are cross-linked to protein G sepharose beads prior to incubation with tissue lysate from the same patient; (**b**) Autoantibodies that are indicative of cancer decline upon treatment (purple antibody) and capture proportionally less autoantigen (purple squares). Eluted fractions are fluorescently labelled and subjected to 2-DE (DIGE). Protein spots that are only immunoprecipitated by the pre operative antibody pool (red spots) or those with a greater intensity are excised from the gel and identified by mass spectrometry. DIGE: Differential gel electrophoresis; Ab: Antibody; green spots: autoantigens immunoprecipitated by the post operative antibody pool only; yellow spots: autoantigens immunoprecipitated by both the pre and post operative antibody pool.

In a different approach, Gagnon and colleagues [30] used pre and post operative sera from ovarian cancer patients to identify autoantibodies whose concentration decline after treatment, reflecting decreased tumour load, and therefore potentially having diagnostic value. Firstly, antigens present in the cancer tissue lysates were immunoprecipitated with plasma antibodies from the same patient that were chemically cross-linked to protein-G sepharose beads (Figure 7a). Eluted fractions were then fluorescently labelled and subjected to differential gel electrophoresis (DIGE) analysis (Figure 7b). Autoantigens that were present at a higher intensity in pre operative samples than post operative samples on the DIGE gel were indicative of a change in autoantibody response due to treatment. By means of a two-dimensional differential gel electrophoresis analysis of immuno-precipitated tumour antigens (2D-DITA) Gagnon *et al.* identified autoantigen S100A7 to have the greatest differential signal [30].

The prevalence of S100A7 autoantibodies in ovarian cancer patients, patients with benign gynaecological disease and healthy controls was analysed by ELISA. The mean plasma levels of S100A7 autoantibodies were significantly greater in both early stage and advanced stage ovarian cancer patients when compared to healthy controls. However, when analysing the mean plasma S100A7 autoantibody levels of cancer patients *versus* those with benign ovarian disease, only patients with advanced cancer had higher mean S100A7 autoantibody levels. Furthermore, analysis of S100A7 autoantibodies alone or as a composite biomarker to CA125 showed that the sensitivity and specificity remained inferior to CA125 alone at detecting ovarian cancer. However, the authors concluded that further analyses in a larger patient cohort were required to determine the real diagnostic potential of S100A7 autoantibodies.

Similarly, Kim and co-workers employed 2D-DITA to analyse the sera from fourteen serous ovarian cancer patients before and after treatment for autoantibodies [31]. From the 36 differential proteins identified in the DIGE gels, stress-induced phosphoprotein-1 (STIP-1) had a 1.16-fold higher expression in pre treatment sera compared to post treatment sera. Noted as having one of the highest differential signals, the stimulation of a humoral immune response by STIP-1 was assessed in 68 ovarian cancer patients (stages unknown), patients with borderline ovarian tumours (*n* = 13) and healthy controls (*n* = 63) by ELISA. The mean STIP-1 concentration in the sera of ovarian cancer patients was significantly different to healthy controls. These results suggest a potential value of STIP-1 autoantibodies as a biomarker for ovarian cancer; however, the relevance for detecting early stage disease remains unclear.

## 3. Conclusion

Novel biomarkers are urgently required to detect early ovarian cancer and reduce the current mortality rate. Evidence suggests that a single biomarker is not sufficiently sensitive or specific for implementation of a global screening strategy, thus a panel of cancer specific biomarkers will need to be generated. Autoantibodies have potential as early diagnostic markers for disease, which has been demonstrated for ovarian and other cancers. The presence of autoantibodies during early stages of malignancy and their inherent stability in sera would enable the detection of cancer at a stage when treatments are most effective. Our recent studies have employed immunoprecipitation and 2D-DITA to identify autoantibodies specific for ovarian cancer. We are now investigating autoantibodies that recognise linear or conformational epitopes of ovarian TAAs. The coupling of immunoaffinity chromatography with existing DIGE technology and SERPA aims to broaden the scope of autoantigens that can be detected. Thus, this approach has the potential to yield novel autoantibodies that can be used for early detection of ovarian cancer. Furthermore, validation of autoantibody biomarkers is a vital step towards clinical implementation. However there is currently a disconnect between the discovery stage and validation which comes through in a number of studies cited in this review.

## Figures and Tables

**Table 1:** Identified ovarian cancer autoantibodies and applied technique.

Technique	Antigen Source (cDNA Library)	Autoantibody Source	TAA	Ref.
**SEREX**	Ovarian cancer cell line Pool of ovarian tumour lysates Normal human testes	Serum	p53 NY-ESO-1 Topoisomerase IIα Ubiquilin-1 Homeobox B6 HMBA inducible protein HDCMA 18P protein	[19]
	Commercial cDNA library	Ascites	HSP90	[20]
	Commercial cDNA library	Serum	IL-8	[21]
	Ovarian cancer tumour lysate	Serum	OVA66	[22]
**Phage Display**	Random peptide library	Ascites	Mimic peptide CVPELGHEC	[24]
	Ovarian cancer cell line	Serum	RCAS1 Signal recognition protein-19 AHNAK-related sequence NASP Nijmegen breakage syndrome 1 Ribosomal protein L4 Homo Sapiens KIAA0419 gene product Eukaryotic initiation factor 5A Casein kinase II Chromodomain helicase DNA-binding protein 1	[25]
**Protein Microarray**	ProtoArray (V3.0)	Serum	94 Autoantigens	[26]
	ProtoArray (V4.0)	Ascites	L-aminoadipate-semialdehyde dehydrogenase-phosphopantetheinyl transferase (AASDHPPT)	[27]
	Ovarian cancer cell line (exosome derived) dot blot array	Serum	Nucleophosmin Cathepsin D SSX common antigen GRP78 P53 Placental-type alkaline phosphatase Heat shock protein 90 NY-ESO-1 Survivin TAG72 CA125 HoxA7	[28]
**Immunoaffinity Purification Methods**	Ovarian adenocarcinoma cell line lysate	Serum	A-kinase anchor protein 9 Eukaryotic translation initiation factor 4γ Midasian RAD50 Talin1 Vinculin Vimention Centrosome-associated protein 350	[29]
	Malignant ovarian tissue lysate	Plasma	S100A7	[30]
	Ovarian tissue lysate	Serum	Stress-induced phosphoprotein-1	[31]

## Abbreviations

2-DE: two-dimensional electrophoresis
2D-DITA: two-dimensional differential gel electrophoresis analysis of immuno-precipitated tumour antigens
DIGE: differential gel electrophoresis
ELISA: enzyme-linked immunosorbent assay
EST: expressed sequence tag
FIGO: federation of gynecologists & obstetricians
LC: liquid chromatography
MS: mass spectrometry
SEPRA: serological proteome analysis
SEREX: Serological analysis of recombinant cDNA expression libraries
TVU: transvaginal ultrasonography
TAA: tumour associated antigen
PPV: positive predictive value
PTM: post translational modification
